# Detection of cancer epigenetic alterations in a pilot cohort of workers exposed to TiO_2_

**DOI:** 10.3389/ftox.2026.1901119

**Published:** 2026-09-22

**Authors:** Eleonora Loi, Loredana Moi, Francesca Floris, Antonietta Marongiu, Paolo Valera, Matteo Serra, Enrico Sanjust, Cesare Zavattari, Alessandro Tommasi, Giuseppe Prencipe, Fabio Boccuni, Rocco Gibilras, Maddalena Papacchini, Marco Di Basilio, Patrizia Zavattari

**Affiliations:** 1 Department of Biomedical Sciences, Unit of Biology and Genetics, University of Cagliari, Cagliari, Italy; 2 Department of Civil-Environmental Engineering and Architecture, University of Cagliari, Cagliari, Italy; 3 National Research Council of Italy, Institute of Environmental Geology and Geoengineering (IGAG), Cagliari, Italy; 4 Department of Biomedical Sciences, Unit of Biochemistry, University of Cagliari, Cagliari, Italy; 5 Department of Computer Science, University of Pisa, Pisa, Italy; 6 Epidemiology and Hygiene, Department of Occupational and Environmental Medicine, National Institute for Insurance against Accidents at Work, Rome, Italy; 7 Department of Technological Innovations and Safety of Plants, Products and Anthropic Settlements, National Institute for Insurance against Accidents at Work, Monte Porzio Catone, Rome, Italy

**Keywords:** colorectal cancer, DNA methylation, GRIA4, job categories, lung cancer, nanoparticles, occupational exposure, TiO_2_

## Abstract

**Introduction:**

The extensive use of TiO_2_ for several applications poses the problem of its safety. Notably, TiO_2_ has been classified as type Group 2 B carcinogen by IARC in 2010. It is widely known that DNA methylation is influenced by the exposure to environmental triggers, including occupational risk factors. The aim of this study was to evaluate the presence of a cancer DNA methylation alteration in TiO_2_ exposed workers from a TiO_2_ powder production plant.

**Methods:**

Due to the possible entry route, by ingestion or by inhalation, of TiO_2_ nanoparticles in the workers of our study cohort, we analysed a DNA methylation biomarker altered in lung and colorectal cancers. The analysis was conducted by MethyLight expressing DNA methylation values as Ct (lower Ct indicates higher methylation level and viceversa), in cfDNA from plasma samples comparing workers with different duties from a plant producing TiO_2_ pigment powder, unexposed subjects as healthy controls and colorectal cancer patients as positive controls, also considering job-related variables.

**Results:**

The obtained results showed that exposed workers displayed higher methylation compared to healthy controls approaching the methylation patterns of colorectal cancer patients, even more evident for workers of some job categories. Moreover, the methylation data would highlight the importance of personal protective equipment, as observed in workers using a gas mask exhibiting a negligible methylation value compared to the job category average methylation level.

**Discussion:**

This preliminary investigation suggests the harmful role of TiO_2_ in altering DNA methylation patterns potentially leading to tumorigenesis and highlights the importance of monitoring workers exposed to TiO_2_ particles.

## Graphical Abstract

**Figure:**
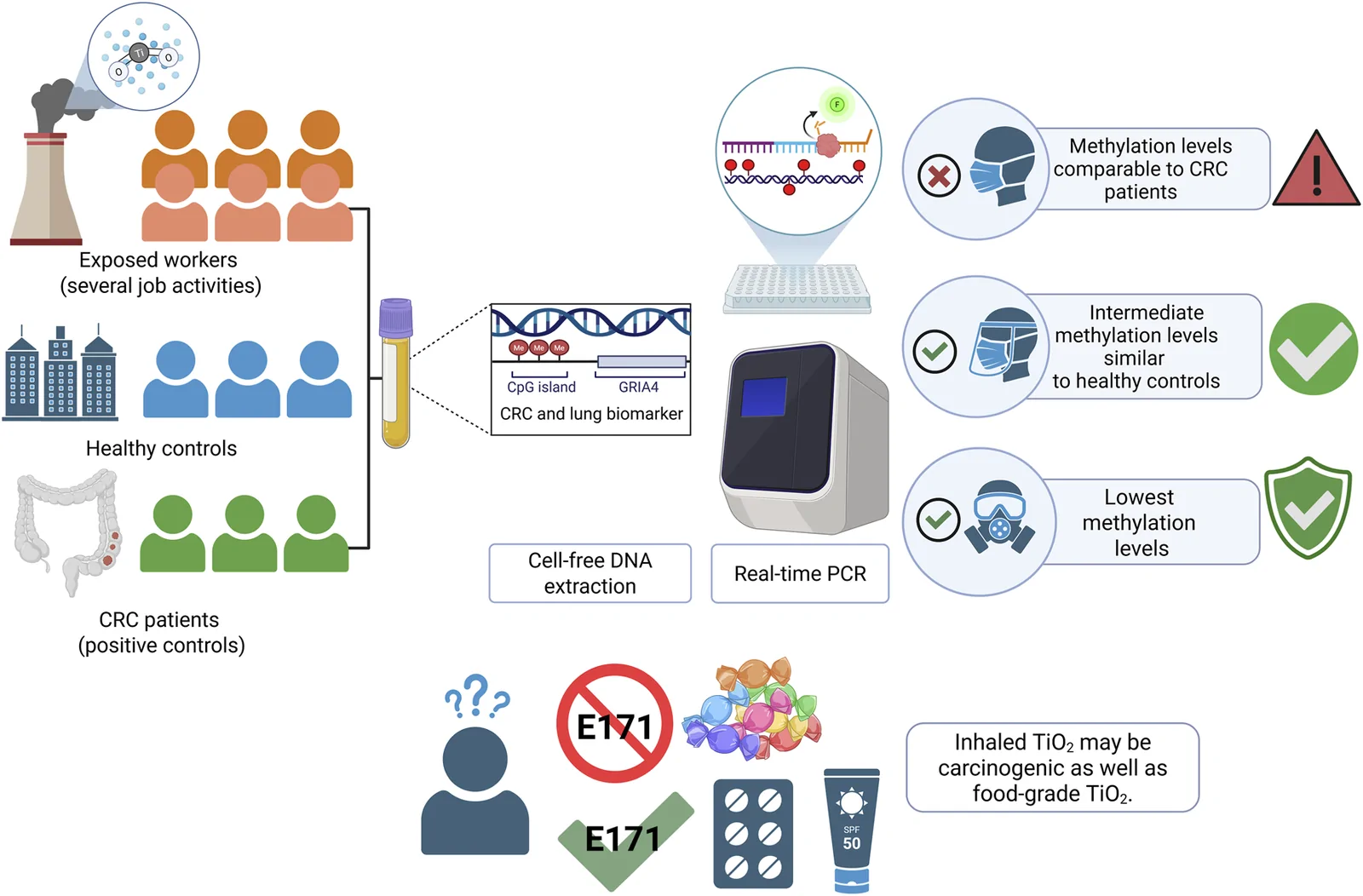

## 1 Introduction

It is widely known that human health could be affected by environmental and genetic factors playing a significant role in the development of diseases (Bollati and Baccarelli, 2010). The interplay between genes and the environment is mediated by epigenetic changes consisting in modification of DNA (e.g., by methylation) or of proteins that intimately associate with DNA, without altering the underlying DNA sequence (Kulis and Esteller, 2010). In the mammalian genome, DNA methylation is the covalent modification of the fifth carbon (C5) of cytosines in the CpG dinucleotides, often clustered in regions called CpG islands (CGIs), catalysed by DNA-methyltransferases (DNMTs) (Kulis and Esteller, 2010). DNA methylation is crucial for development and physiological cell functioning, therefore any defect in this process may lead to several diseases such as cancer characterized by early epigenetic abnormalities (Kulis and Esteller, 2010; Fadda et al., 2018; Loi et al., 2019; Sahu, 2023). Exogenous factors such as work environments, lifestyle, quality of the air, pollutants, exposure to nanoparticles, pharmacological treatments, and others can lead to epigenetic changes affecting human health (Aguilera et al., 2010; Kanherkar et al., 2014). Of note, these changes are not permanent but reversible and for this reason it is possible to re-establish the original condition (Stein, 2012). Since epigenetic changes are early events in cancer they are used as predictive and diagnostic biomarkers (Antonelli et al., 2018; Fadda et al., 2018; Loi et al., 2022; Vega-Benedetti et al., 2022). Moreover, they can also be searched in biological matrices like blood, stool, urine avoiding invasive analysis like biopsy (Kim et al., 2009; Kulis and Esteller, 2010; Fadda et al., 2018; Vega-Benedetti et al., 2022).

The study of epigenetic alterations has also a particular relevance in understanding the effect of occupational exposures to pollutants and new materials, such as titanium dioxide (TiO_2_), which are promising candidates for numerous applications in consumer products, but with potential toxic effects (Buonaurio et al., 2020). TiO_2_ has been widely used for its white colouring properties in several everyday items, including paint, plastics, food, cosmetics, and pharmaceuticals (Waghmode et al., 2019; Cavallo et al., 2023). There are distinct types of TiO_2_ nanostructures, such as nanoparticles (NPs), nanofibers, nanotubes, nanowires, and nano-coatings (Sahu, 2023). NPs are defined as engineered produced particles with a dimension between 1 and 100 nm (Auffan et al., 2009). Previous works have highlighted the association of the exposure to nanomaterial and nanoparticles with epigenetic changes, in particular with DNA methylation (Ravegnini et al., 2015; Pogribna and Hammons, 2021; Valente et al., 2023). Regarding to TiO_2_ exposure, *in vitro* and *in vivo* studies identified DNA methylation alterations in genes involved in cell proliferation, cancer and cellular stress (Ma et al., 2019; Pogribna et al., 2020; Habl et al., 2024; IARC, n. d.). Of note, TiO_2_ was classified as possibly carcinogenic to humans (type Group 2 B) by the International Agency for Research on Cancer (IARC) in 2006, however this evidence was judged as inadequate in humans but sufficient in animal studies (IARC, 2010) (IARC, n. d.). It is also important to consider the size of NPs causing rapid internalization, diffusion and their interaction with cellular organelles and macromolecules such as nucleic acids, inducing genotoxicity. The correlation between genotoxicity and cancer is well established (Kumar et al., 2011). Previous studies have evaluated some aspects of the TiO_2_ potential toxicity in workers of a plant producing TiO_2_ pigment powder. One measured urinary biomarkers of oxidative stress in the workers exposed to rutile powder compared to unexposed subjects finding a dysregulation of Al, Cd, Cr, Cu, Fe, Mn, Pb, Ti, and Zr, and of oxidative stress biomarkers after the working shift (Buonaurio et al., 2020). In another work, the investigation focused on whether the TiO_2_ production process can lead to genotoxicity and cytotoxicity, finding an increase in genotoxicity parameters in workers compared to negative controls. The results of this study also demonstrated that TiO_2_ can overcome both the epithelial cell plasma membrane and accumulate inside the cells leading to apoptosis, but also the nuclear membrane, interacting then with DNA (Ursini et al., 2021). Similarly, studies performed on a different cohort of 35 workers involved in pigment-grade TiO_2_ production investigated the toxicity of long-term exposure to TiO_2_ through the collection of exhaled breath condensate and urine samples and genotoxicity in buccal cells. An alteration in the expression of pro- and anti-inflammatory cytokines and other proteins with a protective role in the lung has been observed in workers’ samples compared to controls as well as DNA damage (Bonetta et al., 2024).

Of note, respiratory diseases, such as bronchial asthma are more common in exposed workers than in controls, however the difference is not statistically significant (Bergamaschi et al., 2022; Cavallo et al., 2023). Furthermore, a relationship between occupational exposure and initial effects on workers’ health, such as respiratory symptoms was demonstrated (Bonetta et al., 2024). Another study evaluated the carcinogenic effect of inhaled TiO_2_ finding a bronchioloalveolar tumour in male rats (Baan, 2007). Growing evidence suggests that TiO_2_ exposure may induce adverse biological effects and its correlation to respiratory diseases but the inhalation of NPs may harm several organs, other than the lung, such as brain, liver, kidney, breast and gastrointestinal tract (Xuan et al., 2023).

Interestingly, another possible entry route of TiO_2_ NPs into the organism is by ingestion affecting the gastrointestinal tract. Notably, it has been reported that colon of rats exposed to TiO_2_ ingestion for 100 days showed pre-neoplastic lesions (Barreau et al., 2021). In fact, it has been shown titanium enters the bloodstream *via* the proximal part of the small intestine and the distal part of the ileum (Pele et al., 2015). Considering these evidence, TiO_2_ exposed workers might have a high risk of NPs inhalation and ingestion possibly leading to the development of cancers like lung and colon ones.

As said above, the exposure to harmful environmental factors represent a potential trigger for early DNA methylation alterations involved in the process of carcinogenesis, detectable even 10 years before cancer diagnosis (Kulis and Esteller, 2010; Stein, 2012; Chappell et al., 2016). Therefore, we decided to investigate the presence of a methylation alteration (associated to *GRIA4*) clearly acting as signature of a cancer state as demonstrated by our group (Barault et al., 2018; Fadda et al., 2018; Vega-Benedetti et al., 2020) and confirmed in other studies (Xi and Zhang, 2017; Sun et al., 2020; Deger et al., 2021; Lukacova et al., 2023; Brenne et al., 2024), in the plasma samples from the same workers analysed in previous studies (Buonaurio et al., 2020; Ursini et al., 2021; Cavallo et al., 2023), compared to the plasma of cancer patients and of unexposed subjects. DNA methylation levels were quantified by MethyLight as a gold standard method for detecting methylation of single loci. To the best of our knowledge, this is the first study that investigates the potential association between occupational exposure to TiO_2_ and a higher cancer risk monitoring aberrant DNA methylation patterns in circulating DNA, suitable for the analysis of such alterations.

## 2 Materials and methods

### 2.1 Study population and sampling

Blood draws were obtained from 47 employees in a TiO_2_, pigment and additives production plant. To avoid interfering with workers operations, blood samples were collected in the morning at the start of the work shift.18 healthy subjects non occupationally exposed to TiO_2_ were volunteers from the same town where the production plant is located and will be referred as “healthy controls” from now on. Their job category is reported in Supplementary Table S1. The study cohort has the following composition: 5 female workers and 42 male workers, 2 female controls and 16 male controls. The average workers age is 42.1 years, while the average controls age is 39.8 years. Among the 47 workers, 41 are directly exposed to TiO_2_, whereas the remaining 6 are employees of the administrative office (OV), however their offices are near the production site. The workers perform different activities, there are 4 bagging area (BA) operators, 6 industrial cleaners (IC), 9 mobile operators (MO), 13 maintainers (MTZ), and 9 white area (WA) operators. Both control group and exposed group filled a questionnaire. The form consists of several items regarding exposure to TiO_2_ and other substances (such as chemicals and cigarette smoke), job and company seniority, the use of PPEs, diet and medical history. The characteristics of the study cohort are summarized in Table 1 and described in detail in Supplementary Table S1 containing the relevant information of the questionnaire.

**TABLE 1:** Characteristics of the study cohort.

Characteristics	Individual information	Total (n = 65)	All workers (n = 47)	Controls (n = 18)
Individual related	Gender	M = 89.2% (n = 58)	M = 89.4% (n = 42)	M = 88.9% (n = 16)
		F = 10.8% (n = 7)	F = 10.6% (n = 5)	F = 11.1% (n = 2)
	Age	42.2 ± 10.4 (n = 65)	42.9 ± 9.5 (n = 47)	40.3 ± 12.5 (n = 18)
	Smoke habits	S = 32.3% (n = 21)	S = 31.9% (n = 15)	S = 33.3% (n = 6)
		NS = 50.8% (n = 33)	NS = 51.1% (n = 24)	NS = 50% (n = 9)
		PS = 16.9% (n = 11)	PS = 17% (n = 8)	PS = 16.7% (n = 3)
	Job seniority	10.8 + 9.1 (n = 60) N/A (n = 5)	10.0 + 8.8 (n = 46) N/A (n = 1)	13.6 + 10.1 (n = 14) N/A (n = 4)
	Company seniority	14.4 ± 10.1 (n = 59) N/A = 7	14.5 ± 10.0 (n = 46) N/A = 1	13.9 ± 10.8 (n = 12) N/A = 6
	Chemical exposure	Y = 18.5% (n = 12)	Y = 21.3% (n = 10)	Y = 11.1% (n = 2)
		N = 81.5% (n = 53)	N = 78.8% (n = 37)	N = 88.9% (n = 16)
Job related	Dust exposure	Y = 7.7% (n = 5)	Y = 10.6% (n = 5)	Y = 0% (n = 0)
		N = 92.3% (n = 60)	N = 89.4% (n = 42)	N = 100% (n = 18)

* CRC, patients are not included, abbreviations: M = male, F = female, S = smoker; NS, no smoker; PS, previous smoker, Y = yes, N = no, N/A = not available data.

Samples from 19 colorectal cancer patients are considered as “positive controls” since our previous study has demonstrated that *GRIA4* methylation is detectable in non-invasive matrices from colorectal cancer patients (Barault et al., 2018; Fadda et al., 2018; Vega-Benedetti et al., 2020; 2022).

Whole blood samples were processed within 3 h after collection. Samples were centrifuged at 1,600 x g for 10 min for phase separation and the collected plasma was submitted to a second centrifugation step at 3,000 x g for 10 min to remove platelets and cell debris. The collected plasma samples were frozen at −80 °C until the extraction. Samples were thawed, centrifuged for 3 min at 11,000 x g, and the supernatant was used for the DNA extraction.

### 2.2 Production plant air monitoring

Exposure measurement campaigns were carried out by National Institute for Insurance against Accidents at Work, in three relevant areas (bagging area, office area and outside ambient) of the production plant. It emerged that workers are mostly exposed to MPs (0.25-micron diameter), however 15% consists of NPs. Air monitoring focused on two types of materials produced in the plant, RXL and TR92, i.e., two titanium dioxide rutile pigments. The monitoring reveals that in both bagging and office area there is TiO_2_ airborne particulate with D_avg_ (average diameter) lower than 100 nm. In the bagging area particulate matter (PM) is more concentrated than the office area, there is also a fraction composed of NPs. In particular, the bagging area has PM10, PM2.5, PM1 concentration two-three times higher than office area and outside ambient. Furthermore, personal measuring device was applied to some workers to monitor TiO_2_ exposure during the 8-h work shift. This monitoring has highly variable values with peaks above 100,000 part/cm^3^ in the BA category. Regarding particles dimension, D_avg_ values outside and in the office area is similar (50 nm), while D_avg_ calculated with the personal measuring device is lower than 300 nm.

### 2.3 DNA methylation analysis

DNA was extracted from 1 mL of plasma, following the protocol of NucleoSpin® Plasma XS kit (Macherey-Nagel GmbH and Co. KG). The EZ DNA-methylation Gold™ kit (Zymo-Research, Irvine, CA, United States) was used to bisulfite convert DNA following the manufacturer’s protocol. To quantify *GRIA4* methylation, a MethyLight qPCR analysis was performed in a final volume of 30 μL including 1xTaqMan Genotyping Master mix (Applied Biosystems, Foster City, CA, United States), 900 nM of each primer, 250 nM of the probe and 50 ng of bisulfite converted DNA. Sequences of the methylation assay are: 5′-GGG​TTG​GTG​TAG​GTT​TGT​T-3’ (primer forward), 5′-CTC​CCC​CCT​TAC​TTT​CTC​ACA​TAC​ACA​CAA-3’ (primer reverse), 5′-AAC​GCC​GCG​ACC​GCC​ACA​C-3’ (probe). Each reaction was conducted on technical duplicate on a DNA Engine Opticon 2 Real-Time Cycler (Bio-Rad, Hercules, CA, United States), the PCR thermal conditions are: initial activation step at 95 °C for 10 min, subsequent 50 cycles of denaturation step at 95 °C for 15 s and annealing/extension step at 60 °C for 1 min. *GRIA4* methylation levels were quantified as average Ct values of the two technical replicates. Lower Ct indicates higher methylation level and *vice versa*. Samples showing Ct values higher than 40 were considered negative. Ct values of 50 were conventionally assigned to samples without a curve output.

### 2.4 Publicly available datasets

We consult *GRIA4* methylation data from TCGA colon adenocarcinoma (COAD) and lung adenocarcinoma (LUAD) datasets by using Wanderer online tool (Díez-Villanueva et al., 2015) and UCSC Xena (Goldman et al., 2020).

### 2.5 Statistical analysis and graphical visualization

A preliminary power analysis was conducted to ensure sufficient statistical power for the current study. Based on our previous results, knowing the optimal performance of our biomarker for cancer detection (AUC = 0.97, specificity = 0.98) (Fadda et al., 2018), the sample size was determined using the central limit theorem. With a confidence level of 95% (Z = 1.96), an expected proportion p = 0.98, and a margin of error d = 0.05 the ideal sample size is N = 30.12. Thus, the selected sample size is sufficient to achieve reliability in our findings. Furthermore, although the sample size is not large, it includes all the workers from the factory so it is the larger one that could be collected.

To compare the different groups, average Ct values and standard deviations were calculated and visualized by scatter plot graph. Graph shows Ct values (y-axis) of the various job categories (BA, IC, MO, MTZ, OV, WA), “healthy controls” (CT) and “positive controls” (CRC) (x-axis). The statistical analysis was carried out by unpaired t-test through GraphPad software (GraphPad Prism 10 Statistics Guide - How to: Multiple t tests, 2026).

Ct values of the analyses of all exposed workers and individual categories with job-related variables were included in a regression model. Regression analysis was performed through GraphPad software by using the least square method. Simple linear regression determines the best fit-value for slope and intercept by fitting a straight line through the data. If the slope is positive, Y (in our case Ct values) increases as X (in our case company seniority or job seniority) increases. On the other hand, if the slope is negative, Y decreases as X increases (GraphPad Prism 10 Curve Fitting Guide - Method tab, 2026). The correlation data is expressed as r^2^.

Heatmaps were generated with R ComplexHeatmap package (Gu et al., 2016).

## 3 Results

### 3.1 *GRIA4* methylation in lung and colorectal cancer tissues

As stated in the Introduction, since TiO_2_ inhalation and ingestion could lead to DNA methylation alterations involved in the development of respiratory and gastrointestinal tumours, we evaluated the methylation levels of *GRIA4*, previously identified by our group as a cancer biomarker, in TCGA LUAD and COAD datasets. Figure 1 shows that both lung (A) and colon (B) tumours display hypermethylation compared to normal tissues, strongly evident in the CGI.

**FIGURE 1:**
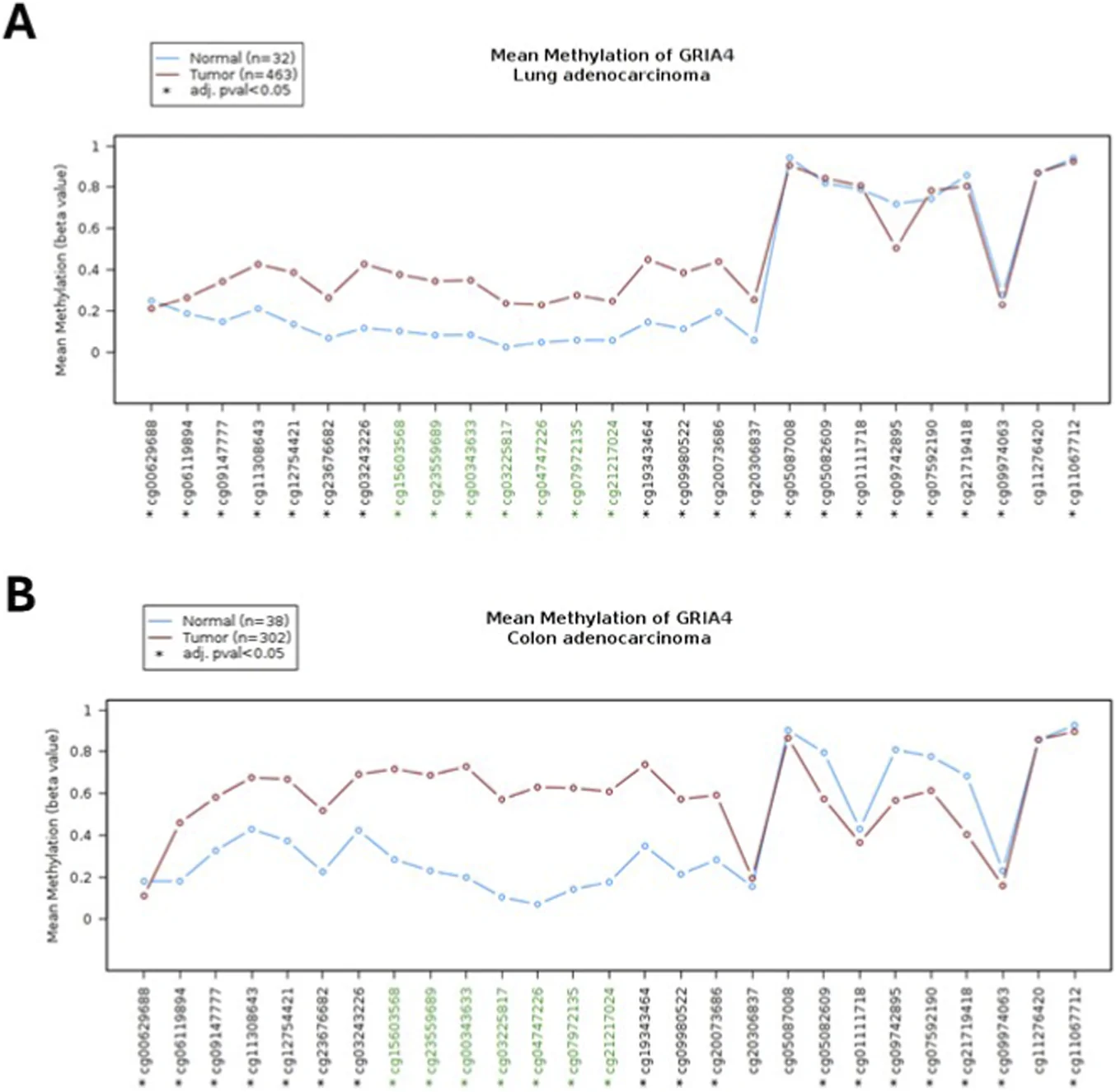
*GRIA4* mean methylation values in lung **(A)** and colon adenocarcinoma **(B)** samples from TCGA 450K Methylation array data. Mean β-values, resulting from the average of the samples (normal indicated with blue dots and tumours indicated with red dots), of each probe. CpG sites in green belong to a CpG island. Pictures were downloaded from Wanderer (http://maplab.imppc.org/wanderer/).

The *in silico* investigations also showed the optimal performance of the biomarker for colon cancer (Supplementary Figure S1) and high specificity for the detection of lung cancer although with a lower sensitivity (Supplementary Figure S2).

### 3.2 *GRIA4* methylation in TiO_2_ exposed workers

The DNA methylation analysis showed that on average, controls (average Ct = 39.7) display low/negligible methylation values compared to the exposed workers (average Ct = 37.4) (p = 0.03) and CRC patients (average Ct = 35.8) (p = 0.0022). The observed higher methylation value of CRC patients was expected considering our previous results allowing to select the alteration as a very informative CRC biomarker. This explains the definition of CRC patients in this study as “positive controls”. As shown in Figure 2, by considering the different job categories, BA and OV showed the most similar levels of methylation to the “positive controls”, followed by WA. These categories showed whereas a marked difference compared to “healthy controls”, although not statistically significant. On the other hand, IC (p = 0.0001) and MO (p = 0.0066) displayed statistically significant lower methylation levels than the “positive controls”. MTZ, OV, WA and “healthy controls” presented some outliers on which we will focus our attention in the discussion. Of note, the identified statistically significant results did not change after the removal of the mentioned outliers but several categories resulted even more similar to CRC, in fact became weakly statistically significant different compared to healthy controls (Supplementary Figure S3).

**FIGURE 2:**
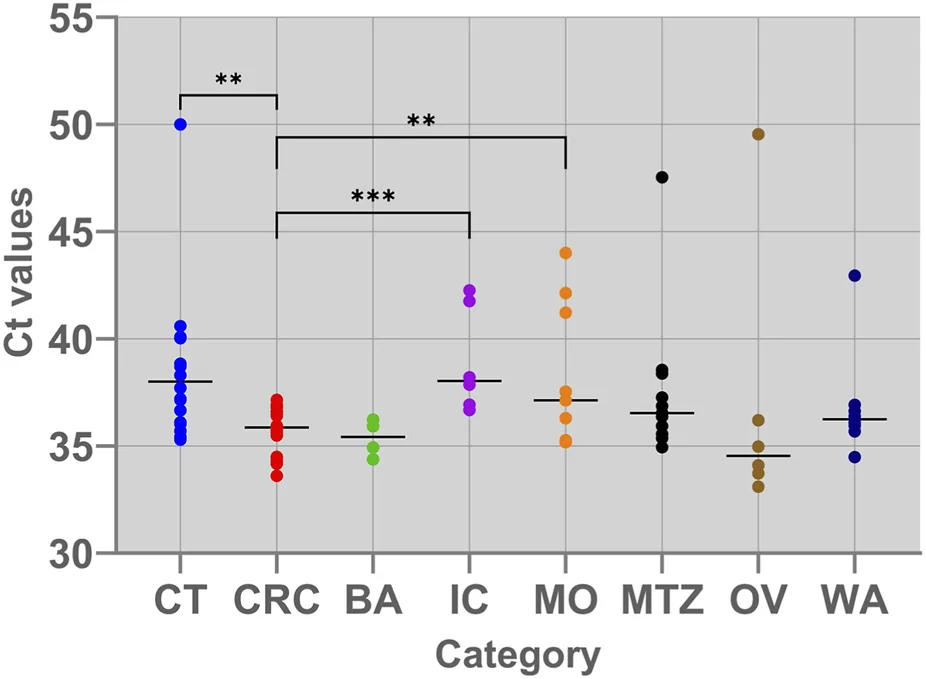
*GRIA4* methylation values, expressed as Ct, in “healthy controls” (CT), “positive controls” (CRC), exposed workers classified into five job categories (BA, IC, MO, MTZ, OV and WA). Every point represents mean Ct value for every subject of the group. The line represents median value. ** indicates statistically significant p < 0.01; *** indicates statistically significant p < 0.001.

Results of the regression analysis are shown in Figure 3. No significant correlation was observed considering all the exposed workers neither for job seniority (A) nor company seniority (B). The analyses performed on the individual job categories showed that only for the OV cohort there was a statistically significant correlation (p = 0.0028) with job seniority (C) but not with company seniority (D).

**FIGURE 3:**
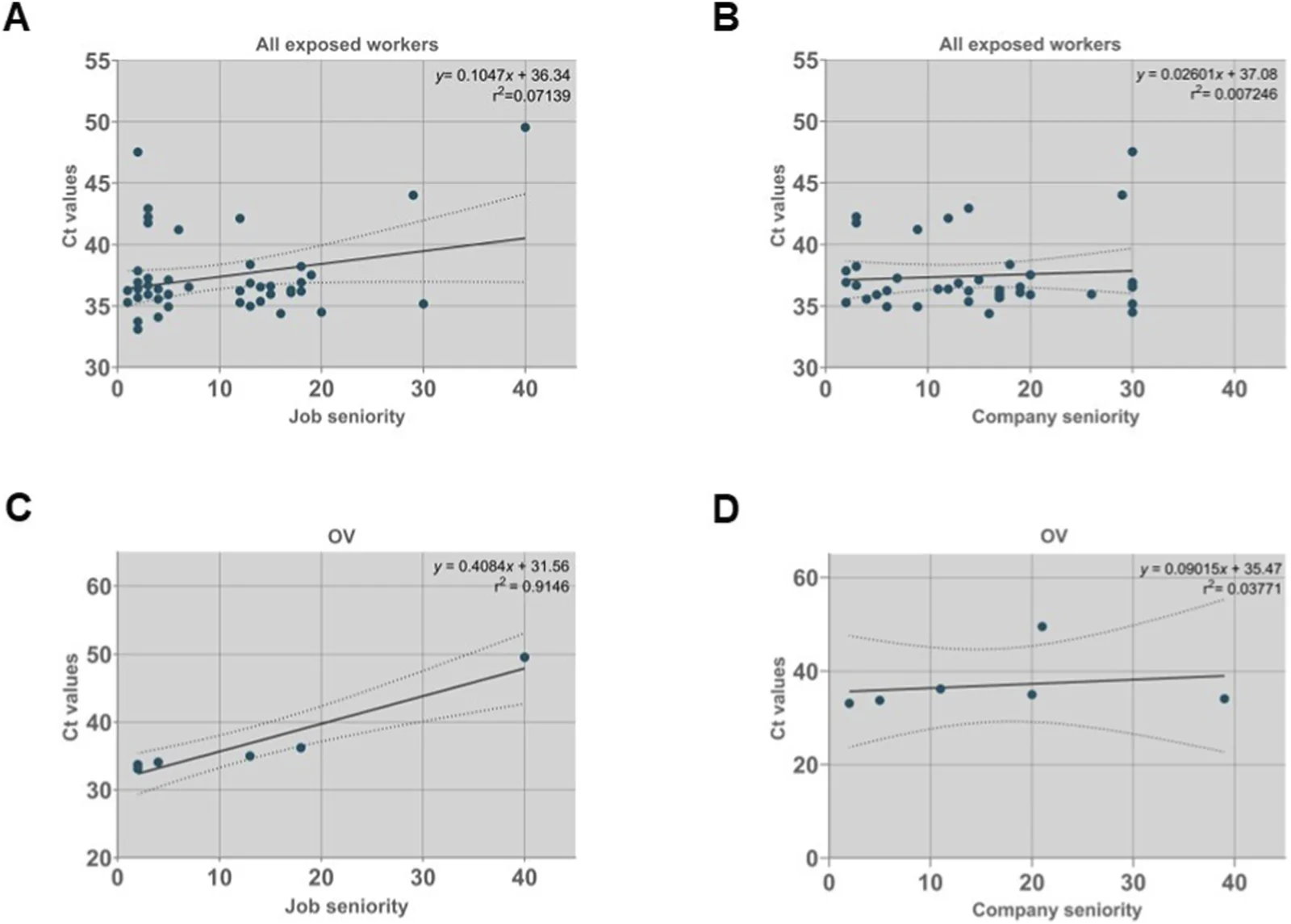
Regression analysis considering job seniority and company seniority (x-axis) against Ct values (y-axis) of all exposed workers (**(A,B)**, respectively) and OV alone (**(C,D)**, respectively).

## 4 Discussion

The present investigation focuses on the potential capacity of TiO_2_ to contribute to an increased cancer risk monitorable by detecting altered DNA methylation patterns, signature of a cancer state, in exposed workers, regardless the mechanism of how TiO_2_ can be involved in the carcinogenic process. TiO_2_ is widely used in several fields for its properties, although studies investigating the effects on human health are proving the toxicity of this compound. Several *in vitro* and *in vivo* studies demonstrated that TiO_2_ can alter numerous physiological functions, however occupational exposure to TiO_2_ is not yet well investigated. Previous research has shown that occupational exposure to rutile dust causes oxidative stress, dysregulation in interleukin release, and DNA damage and that a long-term exposure causes epigenetic adaptation (Halappanavar et al., 2011; Buonaurio et al., 2020; Ursini et al., 2021; Cavallo et al., 2023; Bonetta et al., 2024).

DNA methylation, epigenetic regulator of gene expression known to be affected by xenobiotics, is a cellular mechanism early altered in the carcinogenic process. Few studies have analysed the effect of occupational exposure to dust and fumes on human health through dysregulation of DNA methylation (Van Der Plaat et al., 2019). Altered DNA methylation patterns related to TiO_2_ have been demonstrated through *in vitro* and *in vivo* studies, however most of the research prioritizes the carcinogenic effect of food-grade TiO_2_ (Pele et al., 2015; Setyawati et al., 2018; Barreau et al., 2021). One study conducted on mouse models demonstrated that the severity of the consequences to TiO_2_ dust daily exposure depends on particles dimension (Barreau et al., 2021). In fact, the potential toxicity of TiO_2_ also depends on the chemical form. It is common knowledge that TiO_2_ is chemically inert and unalterable and exhibits distinctive optical properties (high refractive index), and these features are important in relation with its use to obtain bright-white paints. However, such an “inertness” is true for coarse, strongly roasted titania, whereas this could noticeably change when considering micro- or nanoparticles or also hydrous titania (“titanium hydroxide” or also “titanic acid(s)”) obtained through hydrolytic procedures, e.g., by decomposing with acids the alkaline titanates (Gainsford et al., 1995; Waghmode et al., 2019). The European Chemicals agency’s (ECHA) Committee for risk assessment (RAC) in 2021 evaluated TiO_2_ as a carcinogenic compound through inhalation route (H351, Category 2) when supplied on its own or in mixtures with 1% or more of TiO_2_ with an aerodynamic diameter lower than 10 microns (All news - ECHA, 2021).

Although inhalation represents the main route of occupational exposure to TiO_2_ dust, the biological effects of TiO_2_ are not limited to the respiratory system (Proquin et al., 2017; Barreau et al., 2021; Portugal et al., 2024). Previous studies performed in the same cohort of TiO_2_ exposed workers have reported systemic effects associated with occupational exposure (Buonaurio et al., 2020; Ursini et al., 2021; Cavallo et al., 2023), supporting the hypothesis that inhaled TiO_2_ particles may induce biological responses beyond the lungs. Moreover, the workers may accidentally ingest the same TiO_2_ dust since, as reported, they often do not wear appropriate PPEs. It is important to mention that exposure to potentially toxic elements can occur through non-dietary ingestion (Gaberšek and Gosar, 2024; Zupančič et al., 2024). Other exposure routes, such as dietary intake, cannot be completely excluded, as TiO_2_ has been widely used as a food additive; however, their contribution to the biological effects observed in occupationally exposed workers remains difficult to estimate. In this regard, it is also worth mentioning that TiO_2_ has been largely used as food additive because of its anti-caking and colouring properties, hence it may be absorbed through the gastrointestinal tract (Pele et al., 2015; Proquin et al., 2017; Bidu et al., 2025). According to Barreau et al., alimentary TiO_2_ affects DNA repair and carcinogenesis (Barreau et al., 2021).

Considering the previous evidence that NPs have several effects on human health potentially leading to respiratory and gastrointestinal cancers, this study investigated the possible association between TiO_2_ exposure and cancer through the analysis of a tumoral biomarker already identified by our research group and demonstrated to be useful for CRC cancer detection and better definition of prognosis (Barault et al., 2018; Fadda et al., 2018; Vega-Benedetti et al., 2020). The biomarker is an alteration in the DNA methylation pattern of a CpG island located in the promoter region of *GRIA4* (Glutamate Ionotropic Receptor AMP type subunit 4). TCGA data support the usefulness of this biomarker for the detection of both lung and colon cancers. This suggests the interest in analysing *GRIA4* as circulating tumour biomarker in plasma samples from lung cancer patients other than in CRC patients as we have already demonstrated (Barault et al., 2018; Fadda et al., 2018), reinforcing our strategy in the choice of this methylation alteration to be tested in TiO_2_ exposed workers compared to CRC patients as positive controls.

A differential methylation analysis was conducted on DNA extracted from plasma samples of TiO_2_-exposed subjects, “healthy controls”, and CRC patients considered as “positive controls”. The results showed that BA and OV groups display the highest methylation levels comparable to “positive controls”. Interestingly, the evaluation of the air monitoring has shown that BA operators are more exposed to rutile dust compared to the other groups. The apparently unexpected result of the OV group could be explained by several factors: firstly, the close proximity (less than 200 m) of their office to the productive area and the detection of particles with D_avg_ of 50 nm in the office area as well outside, secondly the lack of personal protective equipment (PPE) and thirdly the previous task that some OV carried out for the company. We also considered potential confounding factors such as simultaneous exposure to other toxic substances including smoke, solvents, acids, and basis. Among the exposed subjects, 21.3% are simultaneously exposed to TiO_2_ and chemicals, whereas 31.9% are smokers and 17% previous smokers. Data not shown did not support a contribution of these potential confounding factors to the observed *GRIA4* methylation levels.

Regarding the use of PPE, we noticed some interesting results about higher Ct values (i.e., lower methylation levels) in exposed subjects using PPE with a protective role against rutile dust. Of note, a maintainer worker using a gas mask has a Ct value of 47.53 while the entire MTZ group has an average Ct of 37.4 and the median Ct is even lower (36.5). Another outlier is represented by an office worker covering the task of occupational doctor with a Ct value of 49.54 compared to the average OV Ct value of 36.9. It is important to consider that the occupational doctor carries out his duties at the TiO_2_ production plant occasionally and not daily as the other office workers. Since DNA methylation alterations may arise even 10 years before cancer detection (Loi et al., 2019), it is important to consider that the methylation values recorded in this work may also reflect the previous history of the workers, especially considering that the average job seniority is 10.0 years. In addition, we analysed a correlation between job seniority and Ct values, although a clear association was not found, a slight trend could be observed. Considering the individual job categories, we noticed a statistically significant correlation only between OV Ct values and job seniority, but the Ct value around 50 corresponds to sample of the occupational doctor with a job seniority of 40 years but, as said above, an occasional worker in the plant. We also take into account the correlation with the company seniority, but we did not observe any statistical significance both in the overall cohort as well as in the individual categories.

These results contribute to the increasing knowledge of TiO_2_ potential carcinogenic effect and therefore the need of a strict attention to its use. In 2021, the European Food Safety Authority’s (EFSA) evaluated food additive TiO_2_ (E171) as no longer safe, because it was not possible to establish unequivocally a safe level for daily intake for E171 (EFSA Panel on Food Additives and Flavourings, 2021) (Safety assessment of titanium dioxide as a food additive | EFSA Panel on Food Additives and Flavourings, 2021). The European Medicine Agency (EMA) points out that the feasibility of E171 substitution at this stage is not possible because it may have a negative impact on the quality, safety, and efficacy of medicinal products. For this reason, E171 is still on the list of excipients authorized for use as a colouring agent in medicinal products (Re-evaluation - European Commission, 2010) and is used in many sunscreens for its outstanding ability to either scatter or absorb UV radiation (Gopinath et al., 2020).

In line with its carcinogenic effect, the literature reports that TiO_2_ NPs participate in the epithelial to mesenchymal transition in epithelial cancer cells (Setyawati et al., 2018). Of note, it is known that in different cancer types, genes encoding for adhesion molecules, including cadherins and protocadherins, showed altered DNA methylation patterns (Vega-Benedetti et al., 2019). Moreover, TiO_2_ NPs increased the invasive ability of cancer cells, and it activates TGF-β pathway through mitogen-activated protein kinase (MAPK) pathways, inducing a sustained ROS production in CRC cells (Setyawati et al., 2018). Weichental et al. observed an association between exposure to ultrafine particles (diameter below 0.1 μm) and brain tumour (Weichenthal et al., 2020). The administration *in vivo* of TiO_2_ NPs impairs gut microbiota, creating an imbalance between commensal and pathogenic bacteria, leading to inflammatory reactions caused by oxidative stress. This environment promotes CRC initiation and progression (Kumar et al., 2024).

It is worth mentioning that the aggregation of TiO_2_ NPs may also cause to the formation of fibres that the cells cannot eliminate leading to an accumulation responsible for their toxicity, a mechanism similar to that described for asbestos (Allegri et al., 2016).

Considering the strengths and limitations of our work, we believe that this pilot study can pave the way for an increased attention on the use and exposure to TiO_2_ although the preliminary results derived from the analysis of job categories with a limited number of workers. Another limitation is the size of the healthy control cohort that should match the one of the exposed workers. Moreover, we are aware that several environmental and endogenous factors can confound our results but if considered, they will even decrease the statistical power. Although the difficulty to enrol workers from large companies producing TiO_2_ pigments, we hope that further studies can be carried out on vaster cohorts to validate our findings.

## 5 Conclusion

Our findings contribute to the growing body of evidence indicating that occupational TiO_2_ exposure may be associated with biological changes relevant to carcinogenesis, although further investigations are required to establish causal relationships. The limited sample size requires cautious interpretation but the observation in the technician consistently wearing personal protective equipment may further support the importance of adequate protective measures in reducing occupational exposure. Our findings reveal that occupational TiO_2_ exposure requires attention like it has been for food-grade TiO_2_ additive but not for medicinal products leading to conflicting information for the general population. In fact, the use of TiO_2_ in pharmaceuticals remains debatable despite the ban of E171, because its replacement poses challenges for drug bioavailability due to excipient-excipient or drug-excipient interactions (Panakanti and Narang, 2012). Further studies including larger cohorts, quantitative individual exposure assessment, and detailed physicochemical characterization of TiO_2_ particles are needed to better clarify the relationship between occupational TiO_2_ exposure and the observed biological effects.

## Data Availability

The data that support the findings of this study are included in the Supplementary Material.

## References

[B1] Aguilera O. Fernández A. F. Muñoz A. Fraga M. F. (2010). Epigenetics and environment: a complex relationship. J. Appl. Physiology 109, 243–251. 10.1152/japplphysiol.00068.2010 20378707

[B2] All news - ECHA (2021). Available online at: https://echa.europa.eu/-/titanium-dioxide-proposed-to-be-classified-as-suspected-of-causing-cancer-when-inhaled (Accessed January 9, 2025).

[B3] Allegri M. Bianchi M. G. Chiu M. Varet J. Costa A. L. Ortelli S. (2016). Shape-related toxicity of titanium dioxide nanofibres. PLoS ONE 11, e0151365. 10.1371/journal.pone.0151365 26999274 PMC4801404

[B4] Antonelli M. Fadda A. Loi E. Moi L. Zavattari C. Sulas P. (2018). Integrated DNA methylation analysis identifies topographical and tumoral biomarkers in pilocytic astrocytomas. Oncotarget 9, 13807–13821. 10.18632/oncotarget.24480 29568396 PMC5862617

[B5] Auffan M. Rose J. Bottero J.-Y. Lowry G. V. Jolivet J.-P. Wiesner M. R. (2009). Towards a definition of inorganic nanoparticles from an environmental, health and safety perspective. Nat. Nanotech 4, 634–641. 10.1038/nnano.2009.242 19809453

[B6] Baan R. A. (2007). Carcinogenic hazards from inhaled carbon black, titanium dioxide, and talc not containing asbestos or asbestiform fibers: recent evaluations by an *IARC monographs* working group. Inhal. Toxicol. 19, 213–228. 10.1080/08958370701497903 17886070

[B7] Barault L. Amatu A. Siravegna G. Ponzetti A. Moran S. Cassingena A. (2018). Discovery of methylated circulating DNA biomarkers for comprehensive non-invasive monitoring of treatment response in metastatic colorectal cancer. Gut 67, 1995–2005. 10.1136/gutjnl-2016-313372 28982739 PMC5897187

[B8] Barreau F. Tisseyre C. Ménard S. Ferrand A. Carriere M. (2021). Titanium dioxide particles from the diet: involvement in the genesis of inflammatory bowel diseases and colorectal cancer. Part Fibre Toxicol. 18, 26. 10.1186/s12989-021-00421-2 34330311 PMC8323234

[B9] Bergamaschi E. Bellisario V. Macrì M. Buglisi M. Garzaro G. Squillacioti G. (2022). A biomonitoring pilot study in workers from a paints production plant exposed to pigment-grade titanium dioxide (TiO2). Toxics 10, 171. 10.3390/toxics10040171 35448433 PMC9028136

[B10] Bidu N. S. Reis P. R. D. V. Couto R. D. Fernandes B. J. D. (2025). Is TiO_2_ carcinogenic? A toxicological alert. Toxicol. Ind. Health 41, 07482337251343397–07482337251343413. 10.1177/07482337251343397 40396308

[B11] Bollati V. Baccarelli A. (2010). Environmental epigenetics. Heredity 105, 105–112. 10.1038/hdy.2010.2 20179736 PMC3133724

[B12] Bonetta Sa. Macrì M. Acito M. Villarini M. Moretti M. Bonetta Si. (2024). DNA damage in workers exposed to pigment grade titanium dioxide (TiO2) and association with biomarkers of oxidative stress and inflammation. Environ. Toxicol. Pharmacol. 105, 104328. 10.1016/j.etap.2023.104328 38013010

[B13] Brenne S. S. Madsen P. H. Pedersen I. S. Hveem K. Skorpen F. Krarup H. B. (2024). The prognostic role of circulating tumour DNA detected prior to clinical diagnosis of colorectal cancer in the HUNT study. BMC Cancer 24, 1251. 10.1186/s12885-024-13030-x 39385172 PMC11465842

[B14] Buonaurio F. Astolfi M. L. Canepari S. Di Basilio M. Gibilras R. Mecchia M. (2020). Urinary oxidative stress biomarkers in workers of a titanium dioxide based pigment production plant. IJERPH 17, 9085. 10.3390/ijerph17239085 33291387 PMC7730030

[B15] Cavallo D. Fresegna A. M. Ciervo A. Maiello R. Chiarella P. Buresti G. (2023). Evaluation of systemic genotoxic/oxidative and proinflammatory effects in workers of a titanium dioxide production plant. BioMed Res. Int. 2023, 1–13. 10.1155/2023/7066090 37521120 PMC10386898

[B16] Chappell G. Pogribny I. P. Guyton K. Z. Rusyn I. (2016). Epigenetic alterations induced by genotoxic occupational and environmental human chemical carcinogens: a systematic literature review. Mutat. Research/Reviews Mutat. Res. 768, 27–45. 10.1016/j.mrrev.2016.03.004 27234561 PMC4884606

[B17] Deger T. Boers R. G. De Weerd V. Angus L. Van Der Put M. M. J. Boers J. B. (2021). High-throughput and affordable genome-wide methylation profiling of circulating cell-free DNA by methylated DNA sequencing (MeD-seq) of LpnPI digested fragments. Clin. Epigenet 13, 196. 10.1186/s13148-021-01177-4 34670587 PMC8529776

[B18] Díez-Villanueva A. Mallona I. Peinado M. A. (2015). Wanderer, an interactive viewer to explore DNA methylation and gene expression data in human cancer. Epigenetics and Chromatin 8, 22. 10.1186/s13072-015-0014-8 26113876 PMC4480445

[B19] EFSA Panel on Food Additives and Flavourings (FAF) (2021). Safety assessment of titanium dioxide (E171) as a food additive. EFSA J. 10.2903/j.efsa.2021.6585 33976718 PMC8101360

[B20] Fadda A. Gentilini D. Moi L. Barault L. Leoni V. P. Sulas P. (2018). Colorectal cancer early methylation alterations affect the crosstalk between cell and surrounding environment, tracing a biomarker signature specific for this tumor. Intl J. Cancer 143, 907–920. 10.1002/ijc.31380 29542109

[B21] Gaberšek M. Gosar M. (2024). Oral bioaccessibility of potentially toxic elements in various urban environmental media. Environ. Geochem Health 46, 259. 10.1007/s10653-024-02073-5 38900276 PMC11190014

[B22] Gainsford G. J. Kemmitt T. Lensink C. Milestone N. B. (1995). Isolation and characterization of anionic titanium tris(glycolate) complexes. Inorg. Chem. 34, 746–748. 10.1021/ic00107a029

[B23] Goldman M. J. Craft B. Hastie M. Repečka K. McDade F. Kamath A. (2020). Visualizing and interpreting cancer genomics data via the xena platform. Nat. Biotechnol. 38, 675–678. 10.1038/s41587-020-0546-8 32444850 PMC7386072

[B24] Gopinath K. P. Madhav N. V. Krishnan A. Malolan R. Rangarajan G. (2020). Present applications of titanium dioxide for the photocatalytic removal of pollutants from water: a review. J. Environ. Manag. 270, 110906. 10.1016/j.jenvman.2020.110906gra 32721341

[B25] GraphPad Prism 10 Curve Fitting Guide - Method tab (2026). Available online at: https://www.graphpad.com/guides/prism/latest/curve-fitting/reg_method-tab.htm (Accessed June 6, 2025).

[B26] GraphPad Prism 10 Statistics Guide - How to: Multiple t tests (2026). Available online at: https://www.graphpad.com/guides/prism/latest/statistics/stat_how_to_multiple_t_tests.htm (Accessed July 9, 2025).

[B27] Gu Z. Eils R. Schlesner M. (2016). Complex heatmaps reveal patterns and correlations in multidimensional genomic data. Bioinformatics 32, 2847–2849. 10.1093/bioinformatics/btw313 27207943

[B28] Habl L. El-Sayed M. El Morsi D. Ahmed D. (2024). Titanium dioxide nanoparticles toxicity on DNA methylation: *in vitro* study. Egypt. Soc. Clin. Toxicol. J. 12, 84–96. 10.21608/esctj.2024.280083.1053

[B29] Halappanavar S. Jackson P. Williams A. Jensen K. A. Hougaard K. S. Vogel U. (2011). Pulmonary response to surface‐coated nanotitanium dioxide particles includes induction of acute phase response genes, inflammatory cascades, and changes in microRNAs: a toxicogenomic study. Environ Mol Mutagen 52, 425–439. 10.1002/em.20639 21259345 PMC3210826

[B30] IARC (2010). Carbon black, titanium dioxide, and talc. Available online at: https://publications.iarc.fr/Book-And-Report-Series/Iarc-Monographs-On-The-Identification-Of-Carcinogenic-Hazards-To-Humans/Carbon-Black-Titanium-Dioxide-And-Talc-2010 (Accessed 08, January, 2025). PMC4781574 21449489

[B31] Kanherkar R. R. Bhatia-Dey N. Csoka A. B. (2014). Epigenetics across the human lifespan. Front. Cell Dev. Biol. 2, 49. 10.3389/fcell.2014.00049 25364756 PMC4207041

[B32] Kim J. K. Samaranayake M. Pradhan S. (2009). Epigenetic mechanisms in mammals. Cell. Mol. Life Sci. 66, 596–612. 10.1007/s00018-008-8432-4 18985277 PMC2780668

[B33] Kulis M. Esteller M. (2010). “DNA methylation and cancer,” in Advances in Genetics (Elsevier), 27–56. 10.1016/B978-0-12-380866-0.60002-2 20920744

[B34] Kumar A. Pandey A. K. Singh S. S. Shanker R. Dhawan A. (2011). Cellular uptake and mutagenic potential of metal oxide nanoparticles in bacterial cells. Chemosphere 83, 1124–1132. 10.1016/j.chemosphere.2011.01.025 21310462

[B35] Kumar A. Pramanik J. Batta K. Bamal P. Gaur M. Rustagi S. (2024). Impact of metallic nanoparticles on gut microbiota modulation in colorectal cancer: a review. Cancer Innov. 3, e150. 10.1002/cai2.150 39398260 PMC11467490

[B36] Loi E. Moi L. Fadda A. Satta G. Zucca M. Sanna S. (2019). Methylation alteration of *SHANK1* as a predictive, diagnostic and prognostic biomarker for chronic lymphocytic leukemia. Oncotarget 10, 4987–5002. 10.18632/oncotarget.27080 31452839 PMC6697638

[B37] Loi E. Zavattari C. Tommasi A. Moi L. Canale M. Po A. (2022). HOXD8 hypermethylation as a fully sensitive and specific biomarker for biliary tract cancer detectable in tissue and bile samples. Br. J. Cancer 126, 1783–1794. 10.1038/s41416-022-01738-1 35177798 PMC9174245

[B38] Lukacova E. Burjanivova T. Podlesniy P. Grendar M. Turyova E. Kasubova I. (2023). Hypermethylated GRIA4, a potential biomarker for an early non-invasive detection of metastasis of clinically known colorectal cancer. Front. Oncol. 13, 1205791. 10.3389/fonc.2023.1205791 37476382 PMC10354553

[B39] Ma Y. Guo Y. Ye H. Huang K. Lv Z. Ke Y. (2019). Different effects of titanium dioxide nanoparticles instillation in young and adult mice on DNA methylation related with lung inflammation and fibrosis. Ecotoxicol. Environ. Saf. 176, 1–10. 10.1016/j.ecoenv.2019.03.055 30903973

[B40] Panakanti R. Narang A. S. (2012). Impact of excipient interactions on drug bioavailability from solid dosage forms. Pharm. Res. 29, 2639–2659. 10.1007/s11095-012-0767-8 22610283

[B41] Pele L. C. Thoree V. Bruggraber S. F. Koller D. Thompson R. P. Lomer M. C. (2015). Pharmaceutical/food grade titanium dioxide particles are absorbed into the bloodstream of human volunteers. Part Fibre Toxicol. 12, 26. 10.1186/s12989-015-0101-9 26330118 PMC4557898

[B42] Pogribna M. Hammons G. (2021). Epigenetic effects of nanomaterials and nanoparticles. J. Nanobiotechnol 19, 2. 10.1186/s12951-020-00740-0 33407537 PMC7789336

[B43] Pogribna M. Koonce N. A. Mathew A. Word B. Patri A. K. Lyn-Cook B. (2020). Effect of titanium dioxide nanoparticles on DNA methylation in multiple human cell lines. Nanotoxicology 14, 534–553. 10.1080/17435390.2020.1723730 32031460

[B44] Portugal J. Bedia C. Amato F. Juárez-Facio A. T. Stamatiou R. Lazou A. (2024). Toxicity of airborne nanoparticles: facts and challenges. Environ. Int. 190, 108889. 10.1016/j.envint.2024.108889 39042967

[B45] Proquin H. Rodríguez-Ibarra C. Moonen C. G. J. Urrutia Ortega I. M. Briedé J. J. De Kok T. M. (2017). Titanium dioxide food additive (E171) induces ROS formation and genotoxicity: contribution of micro and nano-sized fractions. MUTAGE 32, 139–149. 10.1093/mutage/gew051 27789654

[B46] Ravegnini G. Sammarini G. Hrelia P. Angelini S. (2015). Key genetic and epigenetic mechanisms in chemical carcinogenesis. Toxicol. Sci. 148, 2–13. 10.1093/toxsci/kfv165 26500287

[B47] Re-evaluation - European Commission (2010). Available online at: https://food.ec.europa.eu/food-safety/food-improvement-agents/additives/re-evaluation_en (Accessed January 8, 2025).

[B48] Sahu S. C. (2023). “Impact of engineered nanomaterials in genomics and epigenomics,” in Impact of Engineered Nanomaterials in Genomics and Epigenomics. Editor Sahu S. C. (Wiley), 1–4. 10.1002/9781119896258.ch1

[B49] Setyawati M. I. Sevencan C. Bay B. H. Xie J. Zhang Y. Demokritou P. (2018). Nano‐TiO _2_ drives epithelial–mesenchymal transition in intestinal epithelial cancer cells. Small 14, 1800922. 10.1002/smll.201800922 29968352

[B50] Stein R. A. (2012). Epigenetics and environmental exposures. J. Epidemiol. Community Health 66, 8–13. 10.1136/jech.2010.130690 22045849

[B51] Sun X. Chen D. Jin Z. Chen T. Lin A. Jin H. (2020). Genome-wide methylation and expression profiling identify methylation-associated genes in colorectal cancer. Epigenomics 12, 19–36. 10.2217/epi-2019-0133 31833403

[B52] Ursini C. L. Di Basilio M. Ciervo A. Fresegna A. M. Maiello R. Buresti G. (2021). Biomonitoring of workers employed in a titanium dioxide production plant: use of buccal micronucleus cytome assay as noninvasive biomarker to evaluate genotoxic and cytotoxic effects. Environ Mol Mutagen 62, 242–251. 10.1002/em.22431 33720463

[B53] Valente A. Vieira L. Silva M. J. Ventura C. (2023). The effect of nanomaterials on DNA methylation: a review. Nanomaterials 13, 1880. 10.3390/nano13121880 37368308 PMC10305477

[B54] Van Der Plaat D. A. Vonk J. M. Terzikhan N. De Jong K. De Vries M. La Bastide-van Gemert S. (2019). Occupational exposure to gases/fumes and mineral dust affect DNA methylation levels of genes regulating expression. Hum. Mol. Genet. 28, 2477–2485. 10.1093/hmg/ddz067 31152171 PMC6643643

[B55] Vega-Benedetti A. F. Loi E. Moi L. Blois S. Fadda A. Antonelli M. (2019). Clustered protocadherins methylation alterations in cancer. Clin. Epigenet 11, 100. 10.1186/s13148-019-0695-0 31288858 PMC6617643

[B56] Vega-Benedetti A. F. Loi E. Moi L. Orrù S. Ziranu P. Pretta A. (2020). Colorectal cancer early detection in stool samples tracing CpG islands methylation alterations affecting gene expression. IJMS 21, 4494. 10.3390/ijms21124494 32599859 PMC7349989

[B57] Vega-Benedetti A. F. Loi E. Moi L. Restivo A. Cabras F. Deidda S. (2022). Colorectal cancer promoter methylation alteration affects the expression of glutamate ionotropic receptor AMPA type subunit 4 alternative isoforms potentially relevant in colon tissue. Hum. Cell 35, 310–319. 10.1007/s13577-021-00640-x 34719006 PMC8732896

[B58] Waghmode M. S. Gunjal A. B. Mulla J. A. Patil N. N. Nawani N. N. (2019). Studies on the titanium dioxide nanoparticles: biosynthesis, applications and remediation. SN Appl. Sci. 1, 310. 10.1007/s42452-019-0337-3

[B59] Weichenthal S. Olaniyan T. Christidis T. Lavigne E. Hatzopoulou M. Van Ryswyk K. (2020). Within-city spatial variations in ambient ultrafine particle concentrations and incident brain tumors in adults. Epidemiology 31, 177–183. 10.1097/EDE.0000000000001137 31714401 PMC7004474

[B60] Xi T. Zhang G. (2017). Epigenetic regulation on the gene expression signature in esophagus adenocarcinoma. Pathology - Res. Pract. 213, 83–88. 10.1016/j.prp.2016.12.007 28049580

[B61] Xuan L. Ju Z. Skonieczna M. Zhou P. Huang R. (2023). Nanoparticles‐induced potential toxicity on human health: applications, toxicity mechanisms, and evaluation models. MedComm 4, e327. 10.1002/mco2.327 37457660 PMC10349198

[B62] Zupančič M. Miler M. Žibret G. (2024). The relationship between the inhalation bioaccessibility of potentially toxic elements in road dust from a heavily polluted industrial area and the source of their pollution. Environ. Pollut. 361, 124810. 10.1016/j.envpol.2024.124810 39181302

