# Supplementary material for: Detection of cancer epigenetic alterations in a pilot cohort of workers exposed to TiO2
Source: Front Toxicol. 2026 Sep 22;8:1901119. doi: 10.3389/ftox.2026.1901119 (PMC13638187; doi:10.3389/ftox.2026.1901119)
Supplement: Supplementary file 1 [file DataSheet1.docx]

Supplementary Material

# 1 Supplementary Figures and Tables

## 1.1 Supplementary Table

**Table S1.** Detailed characteristics of the study cohort.

|  | **Exposed subjects** | **Category** | **Ct values** | **Sex** | **Age** | **Job seniority** | **Company seniority** | **PPE** | **Other risk factors** |
| --- | --- | --- | --- | --- | --- | --- | --- | --- | --- |
| **TiO_2_ workers** | 15B | BA | 34.94 | M | 32 | 5 | 6 | Gloves, glasses, helmet | Drugs |
|  | 16B | BA | 34.38 | M | 47 | 16 | 16 | Helmet, glasses, gloves, shoes, earmuffs, mask | - |
|  | 24B | BA | 35.92 | M | 27 | 5 | 5 | Gloves, glasses, helmet, shoes | Drugs |
|  | 43B | BA | 36.23 | M | 34 | 12 | 14 | Helmet, gloves, mask, glasses, earplugs | Habitation near busy area |
|  | 4B | IC | 36.93 | M | 38 | 2 | 2 | Mask, face shield, coverall, glasses, shoes | Chemicals, cigarette smoke |
|  | 5B | IC | 36.68 | M | 29 | 3 | 3 | - | Chemicals, cigarette smoke, drugs |
|  | 6B | IC | 37.86 | M | 34 | 2 | 2 | Mask, face shield, coverall, glasses, shoes, helmet | Previous exposure to chemicals, drugs |
|  | 10B | IC | 41.77 | M | 26 | 3 | 3 | Mask, face shield, coverall, glasses, shoes, helmet, gloves | Cigarette smoke |
|  | 11B | IC | 42.26 | M | 37 | 3 | 3 | Mask, face shield, coverall, glasses, shoes, helmet, gloves | Chemicals, drugs, previous smoker, habitation near busy area and near gas station |
|  | 12B | IC | 38.22 | F | 54 | 18 | 3 | Helmet, glasses, shoes, gilet | Chemicals, drugs |
|  | 3B | MO | 41.22 | M | 34 | 6 | 9 | Helmet, glasses, gloves, shoes | Drugs |
|  | 7B | MO | 37.13 | M | 40 | 5 | 15 | Helmet, glasses, gloves, shoes, earplugs | Previous exposure to chemicals, drugs |
|  | 8B | MO | 35.27 | F | 28 | 12 | 2 | Helmet, glasses, gloves, mask, shoes | Chemicals, cigarette smoke |
|  | 19B | MO | 44.01 | M | 50 | 29 | 29 | Glasses, helmet, shoes, gloves | Cigarette smoke |
|  | 20B | MO | 37.53 | M | 41 | 19 | 20 | Helmet, gloves, glasses, earmuffs | Chemicals, cigarette smoke |
|  | 26B | MO | 42.14 | M | 45 | 12 | 12 | - | Cigarette smoke |
|  | 27B | MO | 35.17 | M | 60 | 30 | 30 | Helmet, glasses, earmuffs, shoes, acid-resistant clothing | Chemicals, cigarette smoke |
|  | 29B | MO | 36.30 | M | 39 | 17 | 17 | Mask, gloves | Drugs |
|  | 37B | MO | 35.29 | M | 25 | 1 | 2 | Helmet, glasses, gloves, earmuffs, shoes, coverall, earplugs | Chemicals, cigarette smoke, drugs |
|  | 1B | MTZ | 47.53 | M | 49 | 2 | 30 | Helmet, glasses, antigas mask | Chemicals, previous smoker |
|  | 18B | MTZ | 35.37 | M | 47 | 14 | 14 | - | Cigarette smoke |
|  | 22B | MTZ | 35.93 | M | 57 | 3 | 20 | Glasses, gloves, generic PPEs | Cigarette smoke |
|  | 33B | MTZ | 35.56 | M | 40 | 4 | 4 | Helmet, glasses, mask, gloves, earplugs | Chemicals, cigarette smoke, drugs |
|  | 34B | MTZ | 34.94 | M | 37 | 5 | 9 | Helmet, glasses, gloves, earmuffs, shoes | Previous smoker, drugs, habitation near busy area |
|  | 35B | MTZ | 36.38 | M | 49 | 4 | 11 | Helmet, glasses, gloves, earmuffs, shoes, coverall | Drugs |
|  | 36B | MTZ | 36.54 | M | 52 | 7 | 30 | Helmet, glasses, gloves, earmuffs, shoes, coverall, earplug | Drugs |
|  | 38B | MTZ | 36.38 | M | 41 | 13 | 18 | Earmuffs, gloves, seat belts | Cigarette smoke, drugs |
|  | 44B | MTZ | 38.55 | M | 47 | - | - | Helmet, glasses | - |
|  | 45B | MTZ | 36.55 | M | 42 | 14 | 19 | Helmet, glasses | Drugs |
|  | 46B | MTZ | 37.27 | M | 43 | 3 | 7 | Helmet, gloves, mask, glasses | Previous smoker |
|  | 47B | MTZ | 36.86 | M | 41 | 13 | 13 | Helmet, glasses, gloves, shoes | - |
|  | 48B | MTZ | 35.96 | M | 52 | 5 | 26 | Helmet, glasses, gloves, shoes | Chemicals |
|  | 39B | OV | 33.73 | F | 36 | 2 | 5 | - | Chemicals, previous smoker, drugs, habitation near busy area |
|  | 40B | OV | 33.10 | F | 37 | 2 | 2 | - | Drugs |
|  | 41B | OV | 49.54 | M | 64 | 40 | 21 | - | Drugs |
|  | 42B | OV | 34.08 | M | 63 | 4 | 39 | - | Previous smoker, drugs |
|  | NE13B | OV | 34.98 | M | 47 | 13 | 20 | Helmet, glasses, gloves, shoes, earmuffs, mask | Previous exposure to chemicals, previous smoker, drugs |
|  | NE14B | OV | 36.20 | F | 49 | 18 | 11 | - | Cigarette smoke, drugs |
|  | 2B | WA | 36.62 | M | 50 | 15 | 30 | Helmet, glasses, gloves | Drugs |
|  | 9B | WA | 42.95 | M | 35 | 3 | 14 | Helmet, gloves, glasses, earmuffs, earplugs | Chemicals, drugs |
|  | 17B | WA | 36.39 | M | 42 | 2 | 12 | Helmet, gloves, glasses, earplugs | Previous smoker, drugs |
|  | 21B | WA | 36.09 | M | 49 | 17 | 19 | Helmet, glasses, shoes, gloves, earmuffs, mask | Drugs |
|  | 23B | WA | 34.49 | M | 52 | 20 | 30 | Helmet, glasses, earmuffs | Chemicals |
|  | 25B | WA | 35.96 | M | 48 | 15 | 17 | Helmet, glasses, earmuffs, mask | Cigarette smoke |
|  | 28B | WA | 36.92 | M | 53 | 18 | 30 | Earmuffs, mask, shoes | Drugs |
|  | 31B | WA | 36.25 | M | 34 | 1 | 6 | Shoes, coverall | Cigarette smoke |
|  | 32B | WA | 35.68 | M | 41 | 2 | 17 | Helmet, glasses, gloves, earmuffs | Chemicals |
| **Controls** | C1 | Employee | 37.71 | M | 32 | 3 | 1 | - | - |
|  | C2 | Management nurse | 40.08 | M | 49 | 12 | 29 | - | Previous smoker, chemicals |
|  | C3 | Administration collaborator | 40.59 | M | 53 | 10 | 28 | - | Cigarette smoke and drugs |
|  | C4 | Physiotherapist | 40.03 | M | 29 | 5 | 1 | Gloves | Previous exposure to chemicals, smoke and drugs |
|  | C5 | Technical operator | 50.00 | M | 53 | 25 | 25 | - | Previous smoker, drugs |
|  | C6 | Employee | 37.20 | M | - | 15 | 22 | - | Cigarette smoke, drugs |
|  | C7 | Doctor | 38.84 | F | 47 | 13 | 13 | - | - |
|  | C8 | Administration employee | 38.30 | M | 50 | 30 | 20 | - | Drugs |
|  | C9 | Nurse | 35.71 | M | 51 | 23 | 15 | Gloves, glasses | Previous exposure to chemicals and chalk, cigarette smoke, drugs |
|  | C10 | Administration collaborator | 35.42 | M | 44 | 1 | 1 | - | - |
|  | C11 | - | 38.70 | M | 31 | - | - | - | - |
|  | C12 | Doctor | 36.66 | M | 31 | 5 | 5 | - | Drugs |
|  | C13 | Student | 50.00 | M | 26 | - | - | - | - |
|  | C14 | Student | 35.30 | F | 22 | - | - | - | Drugs |
|  | C15 | Car wash operator | 37.15 | M | 34 | 7 | 7 | Gloves, mask | Previous smoker, chemicals and drugs |
|  | C16 | Physiotherapist | 36.03 | M | 33 | 10 | - | - | Cigarette smoke and drugs |
|  | C17 | Student | 50.00 | M | 25 | - | - | - | Cigarette smoke and drugs |
|  | C18 | Doctor | 36.10 | M | 66 | 32 | - | - | - |

BA: Bagging operators; IC: Industrial cleaners; MO: Mobile operators; MTZ: Maintenance operators; OV: Administrative office employees; WA: White area operators; Ct: Threshold cycle; PPE: Personal protective equipment.

## 1.2 Supplementary Figures

**Supplementary Figure 1.** Visualisation of the methylation levels in the CpG island associated to *GRIA4* analysed in our work. Data from TCGA-COAD.

**Supplementary Figure 2.** Visualisation of the methylation levels in the CpG island associated to *GRIA4* analysed in our work. Data from TCGA-LUAD.

.

**Supplementary Figure 3.** *GRIA4* methylation values expressed as Ct values without the outliers in categories CT, MTZ, OV and WA.
